# Genomic Insights into a New *Burkholderia cenocepacia* Sequence Type Linked to Cepacia Syndrome in Cystic Fibrosis

**DOI:** 10.1007/s00284-026-04994-z

**Published:** 2026-06-11

**Authors:** Tatiane S. Xavier, Felipe A. Simão, Heloisa S. Rosa, Jade C. S. Colomer, Renata W. Cohen, Tânia W. Folescu, Ana Paula D’A. Carvalho-Assef, Robson S. Leão, Elizabeth A. Marques

**Affiliations:** 1 Laboratório de Microbiologia da Fibrose Cística, Departamento de Microbiologia, Imunologia e Parasitologia, Faculdade de Ciências Médicas, Universidade do Estado do Rio de Janeiro, Rio de Janeiro, Brazil; 2 Instituto Nacional de Saúde da Mulher, da Criança e do Adolescente Fernandes Figueira, Centro de Referência para Crianças e Adolescentes com Fibrose Cística – Ministério da Saúde, Fundação Oswaldo Cruz, Rio de Janeiro, Brazil; 3 Laboratório de Bacteriologia Aplicada à Saúde Única e Resistência Antimicrobiana, Instituto Oswaldo Cruz, Rio de Janeiro, Brazil

## Abstract

*Burkholderia cenocepacia* is an important pathogen in cystic fibrosis (CF) patients, responsible for chronic lung infections and, in severe cases, cepacia syndrome (CS), characterized by necrotizing pneumonia, bacteremia, and high mortality. Epidemic lineages, such as *B. cenocepacia* J2315 (ET12) and ST32, are highly transmissible and frequently associated with CS. In this study, five *B. cenocepacia* isolates recovered from sputum and blood samples of a pediatric CF patient who developed CS were sequenced and analyzed in silico using multilocus sequence typing (MLST), phylogenomics, genomic similarity metrics, and comparative genomics to identify virulence determinants, resistance genes, and mobile genetic elements. A novel sequence type (ST2424), not associated with any known clonal complex, was identified. Phylogenomic analysis revealed high genetic relatedness among the isolates, consistent with a monoclonal infection undergoing within-host microevolution. Comparative analyses revealed distinct genetic profiles: *aaiB* (T6SS) was present in all isolates, whereas *narG* and *manCcore* were exclusive to bloodstream isolates. Acquired resistance determinants, including *bla*_*OXA−1043*_, *sul1*, *aac(6′)-IIc*, and *aadA1*, were detected and were absent from the reference strains *B. cenocepacia* J2315 and ST32. Mobile genetic elements, including insertion sequences from the IS3, IS5, and IS200/IS605 families, as well as a putatively conjugative plasmid encoding Tra/Trb subunits of the type IV secretion system, were also identified. These findings describe a novel *B. cenocepacia* sequence type associated with fatal CS and highlight the high genomic plasticity of this species, reinforcing the importance of genomic surveillance to detect emerging variants and improve clinical management in CF.

**Supplementary Information:**

The online version contains supplementary material available at 10.1007/s00284-026-04994-z.

## Introduction

*Burkholderia cepacia* complex (Bcc) consists of over 20 phylogenetically related species of non-fermenting Gram-negative bacilli, traditionally categorized into genomovars [1]. In Cystic Fibrosis (CF), chronic infections caused by Bcc are linked to a rapid deterioration in lung function and heightened mortality [2].

Its relevance arises from its intrinsic resistance to multiple antimicrobials, including aminoglycosides and polymyxins, as well as disinfectants, coupled with an exceptional capacity to adapt to a range of ecological niches such as plant rhizospheres, soils, freshwater environments, healthcare systems, and pharmaceutical solutions [3, 4]. Biofilm formation increases persistence in the airways, shielding the pathogen from host immune defenses and standard therapies, thus complicating the clinical management of CF respiratory infections [5].

The Bcc genome is large, ranging from approximately 6 to 10 Mbp, and typically organized into three replicons. Chromosome 1 is conserved and enriched in essential genes, chromosome 2 contains adaptation loci, and the third replicon (megaplasmid pC3) is highly variable and associated with virulence, resistance, and specialized metabolites including antifungal compounds [6, 7]. An estimated 10% of the genome originates from horizontal gene acquisition, contributing to a diverse reservoir of mobile genetic elements [8].

Among Bcc species, *Burkholderia cenocepacia* (lineage IIIA) is particularly associated with accelerated decline in lung function and with cepacia syndrome (CS), an acute necrotizing pneumonia characterized by high fever, bacteremia, and rapidly progressive respiratory failure with high mortality [9]. Although the exact incidence of CS is unclear, with only a few reports published the mortality rates reaching as high as 50% [10].

The pathogenic potential of *B. cenocepacia* is supported by a broad arsenal of virulence factors, including adhesins, invasins, toxins, enzymes, siderophores, and secretion systems, which together facilitate adhesion to host tissues, invasion of cells, and evasion of immune responses [8]. Beyond these mechanisms, *B. cenocepacia* invades macrophages and epithelial cells, persisting by blocking lysosomal fusion and manipulating autophagy. Through the type VI secretion system (T6SS), it interferes with host defenses, and the negative regulation of the autophagic pathway ensures its prolonged survival, resulting in severe inflammation and pulmonary deterioration in CF [11].

Within *B. cenocepacia*, Clonal Complex 31 (CC31) is the lineage most strongly associated with severe CF outbreaks and represents the largest cluster in the multilocus sequence typing (MLST) database [12]. Within subgroup IIIA, the epidemic clone *B. cenocepacia* J2315 (corresponding to ET12 and ST28) and *B. cenocepacia* ST32 are particularly notable for their high transmissibility and virulence. Despite belonging to the same CC31 cluster, they differ in their multilocus sequence typing (MLST) allelic profiles, which may result in phenotypic variations with clinical impact [13, 14].

During chronic infection, clonal evolution can generate genetic variability that shapes disease progression. Comparative genomics is therefore a powerful tool to identify adaptive processes and to improve the understanding of persistence and virulence in these lineages [15, 16].

In this study, we analyzed the genome of five clinical isolates of *B. cenocepacia* obtained from a Brazilian CF pediatric patient with CS, comprising one sputum and four bloodstream isolates. We evaluated the virulence repertoire, resistome, and the presence of mobile genetic elements, comparing them with the epidemic strains *B. cenocepacia* J2315 and *B. cenocepacia* ST32 to contextualize their phylogenomic relationships and identify genomic features potentially associated with persistence and systemic dissemination.

## Materials and Methods

### Sample Collection and Identification

An 11-year-old female patient, followed since infancy at the Cystic Fibrosis Reference Center of the Instituto Nacional de Saúde da Mulher, da Criança e do Adolescente Fernandes Figueira (IFF/Fiocruz, Rio de Janeiro) for recurrent respiratory infections, was diagnosed with CF in 2014 with a homozygous F508del mutation. She developed chronic *Pseudomonas aeruginosa* infection in 2019 and had her first Bcc isolation in 2021. In 2023, her clinical condition deteriorated with multiple hospitalizations consistent with CS, progressing to respiratory failure and death.

The five isolates related to the CS case were obtained during hospitalization and were initially identified by phenotypic methods as belonging to the Bcc. The set comprised one sputum isolate (3424) and four blood isolates (3442, 3443, 3412, and 3415). The sputum isolate was recovered on August 28, 2023, while the blood isolates were collected between September 6 and 10, isolate 3442 on September 6, isolates 3443 and 3412 on September 8, and isolate 3415 on September 10.

### Identification of Isolates by Mass Spectrometry

The isolates were identified by matrix-assisted laser desorption/ionization time-of-flight mass spectrometry (MALDI-TOF MS) using the Microflex LT instrument (Bruker Daltonics, Leipzig, Germany) and FlexControl v3.4 software (Bruker Daltonics).

### Whole-Genome Sequencing and Bioinformatics Analysis

Bacterial DNA was extracted and purified using the QIAamp DNA Mini Kit (QIAGEN, Germany) and quantified with the QuantiFluor^®^ system (Promega, USA). Genomic libraries were prepared with the Nextera XT Kit (Illumina, USA) and sequenced on the Illumina MiSeq platform (Illumina Inc., California, USA).

Read quality was evaluated using FastQC v0.11.9 implemented on the Galaxy Australia platform (https://usegalaxy.org.au) and the sequences were assembled with Unicycler v0.4.8 on the Bacterial and Viral Bioinformatics Resource Center (BV-BRC) platform v3.49.1 (https://www.bv-brc.org). Assembly metrics were calculated using the Quality Assessment Tool for Genome Assemblies (QUAST) v5.3.0 (https://quast.sourceforge.net). Genome assembly quality was assessed using CheckM v1.2.4 (completeness ≥ 90% and contamination < 5%).

Annotation was performed using Rapid Annotation using Subsystems Technology (RAST) v2.0 (https://rast.nmpdr.org) and RASTtk [17] integrated into BV-BRC. Sequence integrity was confirmed using BLAST v2.10.0+ (https://blast.ncbi.nlm.nih.gov/Blast.cgi) with BLASTn and BLASTp algorithms.

Genomovar assignment was performed by in silico analysis of the *recA* gene extracted from the assembled genomes and compared with reference *recA* sequences representative of *B. cenocepacia* genomovar III, subgroups IIIA, IIIB, and IIIC. MLST was performed in silico according to the Bcc scheme available at the PubMLST database (https://pubmlst.org/), based on the allelic profiles of seven housekeeping genes (*atpD*, *gltB*, *gyrB*, *recA*, *lepA*, *phaC*, and *trpB*), with allele assignment and sequence type determination confirmed using the Center for Genomic Epidemiology (CGE) v2.0.9 (https://www.food.dtu.dk).

Species confirmation and taxonomic delimitation were performed using whole-genome-based approaches, including digital DNA–DNA hybridization (dDDH) with the Genome-to-Genome Distance Calculator (GGDC) v3.0 (https://ggdc.dsmz.de), employing formula 2 and BLAST+, applying a ≥ 70% species threshold. Average Nucleotide Identity (ANI) was estimated using OrthoANIu on EzBioCloud (https://www.ezbiocloud.net), and Average Amino Acid Identity (AAI) was calculated using Enveomics (https://enve-omics.ce.gatech.edu), both applying a ≥ 95% threshold.

Taxonomic and phylogenomic placement based on whole-genome sequences was performed using type strains of validly published species with the Type (Strain) Genome Server (TYGS) v405 (https://tygs.dsmz.de). Single-nucleotide polymorphism (SNP)-based phylogenetic analysis was conducted using CSI Phylogeny v1.4 (https://cge.food.dtu.dk/services/CSIPhylogeny/) with default settings. The resulting phylogenetic tree was visualized and edited using Molecular Evolutionary Genetics Analysis (MEGA) v11.0.13. All taxonomic names were verified using the List of Prokaryotic names with Standing in Nomenclature (LPSN) (https://lpsn.dsmz.de), accessed February 4, 2026.

For comparative analysis, the genomes of *B. cenocepacia* J2315 (ST28) (GenBank accession NC_011001.1) and *B. cenocepacia* ST32 (GenBank accession NZ_CP011920.1) were used.

Virulence genes were identified using VFanalyzer (https://www.vfdb.org/analyzer) based on the Virulence Factors Database (VFDB). The resistome was characterized using ResFinder v4.7.2 (https://cge.food.dtu.dk/services/ResFinder) for acquired resistance; Resistance Gene Identifier (RGI) v6.0.3 and the Comprehensive Antibiotic Resistance Database (CARD) v4.0.0 (https://card.mcmaster.ca) for acquired, intrinsic, and variant resistance; and the Antimicrobial Resistance (PATRIC AMR) module [18] from BV-BRC, based on AMRFinderPlus (NCBI), to complement the identification of intrinsic mechanisms, variants, and chromosomal elements.

The mobilome was characterized using PHASTEST v3.0 (https://phastest.ca) to identify prophage regions, and IslandViewer v4 (https://pathogenomics.sfu.ca/islandviewer) with SIGI-HMM and IslandPath-DIMOB to predict pathogenicity islands, using *B. cenocepacia* J2315 as the reference genome. MobileElementFinder v1.0.3 (https://cge.food.dtu.dk/services/MobileElementFinder) was applied to detect insertion sequences and transposable elements; PlasmidFinder v2.1.6 [19] together with MOB-suite v3.1.9 [20] were used to identify and classify plasmids. Putative plasmid contigs were validated by BLASTn and annotated within the BV-BRC platform.

The Whole Genome Shotgun (WGS) sequencing project was deposited in the National Center for Biotechnology Information (NCBI) under the following accession numbers: JBQRTX000000000 (3424, BioProject PRJNA1309077); JBQRTY000000000 (3442, BioProject PRJNA1309082); JBQRTZ000000000 (3443, BioProject PRJNA1309087); JBQRTV000000000 (3412, BioProject PRJNA1309062); and JBQRTW000000000 (3415, BioProject PRJNA1309070).

## Results

### Identification by Mass Spectrometry

MALDI-TOF identification yielded scores ranging from 2.33 to 2.42, consistent with species-level confidence (≥ 2.30). All isolates were identified as *B. cenocepacia*.

### Whole-Genome Sequencing Analysis

Genome assembly quality metrics, including genome size, number of contigs, GC content, coverage, completeness, and contamination, are presented in Table 1.

**Table 1:** Genomic composition data of the five *B. cenocepacia* isolates

	3424	3442	3443	3412	3415
Genome size (bp)	7,546,741	7,736,515	7,716,984	7,745,325	7,663,244
GC content (%)	67	67.5	67.5	67.5	67.5
Number of contigs	164	134	160	122	207
N50 (kb)	87.9	125.5	85.2	111.1	62.4
L50	25	20	23	24	32
Coverage (×)	83	68	39	74	33
CDS (n)	7,490	7,636	7,648	7,628	7,626
RNAs (n)	62	60	60	61	57
Subsystems (n)	389	396	396	396	396
CheckM completeness (%)	96.71	97.74	97.89	97.73	97.34
CheckM contamination (%)	2.78	2.93	3.18	2.84	2.93

bp base pairs; GC guanine–cytosine content; CDS coding DNA sequences; RNAs ribonucleic acids; n: number

All isolates were identified as *B. cenocepacia* genomovar IIIA based on *recA* gene sequence analysis, showing sequence identities ranging from 99.62% to 100.00% when compared with reference accessions.

Allelic profiles of seven housekeeping genes were obtained from the annotated genomes using the PubMLST database. A novel allele in *gltB*, designated *gltB_1015*, was identified, resulting in a new sequence type (ST2424; ID 5285) not assigned to any known clonal complex.

Genomic similarity analyses based on dDDH, ANI, and AAI were performed by comparison with *B. cenocepacia* reference genomes. Relative to strain *B. cenocepacia* J2315, all isolates exhibited dDDH values ≥ 91.50%, ANI ≥ 98.98%, and AAI ≥ 98.19%. When compared with the *B. cenocepacia* ST32 genome, the values were ≥ 88.40%, ≥ 98.77%, and ≥ 97.63%, respectively.

**Fig. 1:**
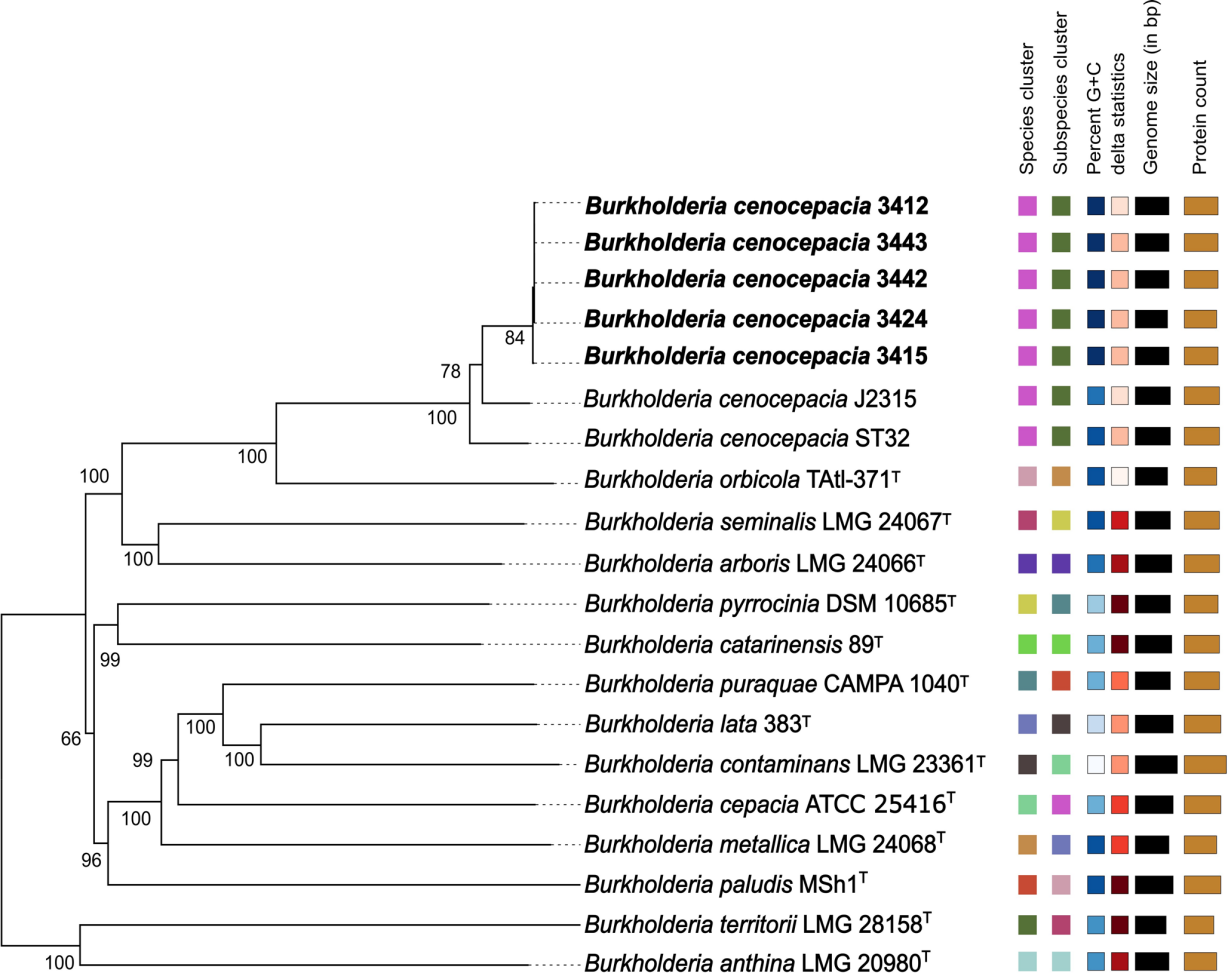
Phylogenetic tree inferred with FastME v2.1.6.1 based on GBDP distances calculated from whole-genome sequences using the Type (Strain) Genome Server (TYGS). The tree shows the phylogenomic relationships among *B. cenocepacia* isolates (3415, 3443, 3412, 3442, and 3424), reference strains (J2315 and ST32), and the closest type strains within the genus *Burkholderia*. Branch lengths are proportional to GBDP distances (d5 formula), and pseudo-bootstrap values (> 60%) are based on 100 replicates, with an average branch support of 80.6%. Leaf labels indicate species and subspecies cluster affiliation, genomic G + C content, delta values (0.132 in this tree) reflecting phylogenetic precision (lower values correspond to higher precision), genome size, and number of proteins

The phylogenomic tree generated by the Type (Strain) Genome Server (TYGS) clustered the five clinical isolates (3415, 3443, 3412, 3442, and 3424) into a single clade supported by an 84% bootstrap value. The color-coded species and subspecies clusters indicated that all analyzed strains belong to the same species and subspecies represented by the reference strains (*B. cenocepacia* J2315 and ST32). Additionally, the isolates grouped phylogenomically in a branch closely related to these reference strains, with 100% bootstrap support, corroborating their affiliation with the *B. cenocepacia* genomovar IIIA lineage (Fig. 1).

**Fig. 2:**
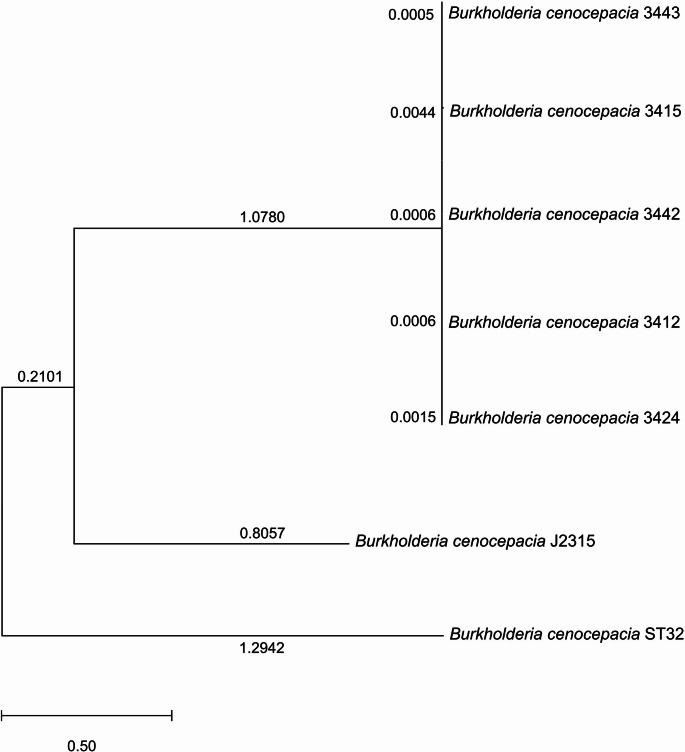
SNP-based phylogenomic tree of clinical *B. cenocepacia* isolates and reference strains. The tree was constructed using SNPs through the CSI Phylogeny platform. The five clinical isolates analyzed (3424, 3442, 3443, 3412, and 3415) form a single clade, distinct from the reference strains *B. cenocepacia* J2315 and *B. cenocepacia* ST32. Branch lengths are proportional to the genetic distance inferred from SNPs. The scale bar indicates the number of substitutions per site

The SNP-based phylogenomic analysis showed that the five *B. cenocepacia* clinical isolates clustered together in a single monophyletic group. Pairwise SNP distances among the isolates ranged from 16 to 253 SNPs. The smallest distances were observed among bloodstream isolates 3442, 3443, and 3412, which differed by 16 to 32 SNPs. The sputum isolate 3424 showed pairwise SNP distances ranging from 82 to 112 SNPs in comparison with the bloodstream isolates. Isolate 3415, recovered from blood, presented higher pairwise SNP distances relative to the other bloodstream isolates, ranging from 183 to 253 SNPs (Fig. 2).

When compared with the reference strains, all clinical isolates exhibited markedly higher SNP distances. Pairwise SNP distances between the clinical isolates and *B. cenocepacia* J2315 ranged from 26,264 to 26,355 SNPs, while distances to *B. cenocepacia* ST32 ranged from 31,811 to 31,878 SNPs. The SNP distance between the reference strains J2315 and ST32 was 29,997 SNPs (Fig. 2).

### Distribution and Comparison of Virulence Genes

In the five *B. cenocepacia* clinical isolates, virulence factors were predicted and compared with those identified in the epidemic strains *B. cenocepacia* J2315 and *B. cenocepacia* ST32. A total of 100 virulence-associated genes were identified and grouped into 11 functional categories according to the VFDB classification, 90 of which formed a conserved core shared by all genomes.

The main categories comprised adhesion (*boaA*/*boaB* and multiple type IV pilus components), antiphagocytosis (the wcb operon), invasion/chemotaxis (*che*, *flg*/*flh*/*fli*, *motA*/*B*, *tsr*), quorum sensing (*bsp*/*pmlI*/*pmlR*), and secretion systems T3SS/T6SS-1 (*aai*, *bsaQ*, *bprA*, *clpV*) (Supplementary Table S1).

Distinct distribution patterns were observed for a subset of genes compared to the epidemic references: the AAI/SCI-II T6SS component *aaiB* was found in all five clinical isolates, while *narG* and *manCcore* were detected exclusively in bloodstream isolates (3442, 3443, 3412, and 3415) (Table 2).

**Table 2:** Putative virulence genes differing among the five clinical isolates of *B. cenocepacia* predicted using the Virulence Factors Database in comparison with the reference strains *B. cenocepacia* J2315 and *B. cenocepacia* ST32

Gene	Product encoded	3424	3442	3443	3412	3415	J2315	ST32
**Secretion system**								
*aaiA*	AAI/SCI-II T6SS	P	P	P	P	P	A	P
*aaiB*		P	P	P	P	P	A	A
**Anaerobic respiration**								
*narG*	Nitrate reductase	A	P	P	P	P	A	A
**Antiphagocytosis**								
*wbjD* */wecB*	Capsular polysaccharide	P	P	P	P	P	P	A
*wcbC*	Capsule I	P	P	P	P	P	P	A
*wcbE*		A	A	A	A	A	A	P
**Immune evasion**								
*manCcore*	LPS	A	P	P	P	P	A	A
**Iron uptake**								
*pchF*	Pyochelin	P	P	P	P	P	A	P
*ccmE* *ccmF*	Cytochrome c maturation locus	A	A	A	A	A	A	P

P present; A absent

### Resistome Analysis

Analysis using ResFinder revealed that the clinical isolates exhibited a homogeneous acquired resistance profile characterized by the presence of the *aac(6′)-IIc*, *aadA1*, and *sul1* genes, which were absent in *B. cenocepacia* J2315 and *B. cenocepacia* ST32.

Moreover, CARD/RGI analysis showed that the clinical isolates harbored the genes *aadA* (aminoglycoside resistance), *bla*_*OXA−1043*_ and *penB-4* (β-lactamases), as well as *qacE∆1* (antiseptic resistance). The *bla*_*OXA−1142*_ (β-lactamase) was identified only in *B. cenocepacia* J2315 (Table 3).

**Table 3:** Antimicrobial resistance genes identified in the five clinical isolates of *B. cenocepacia* and in the reference strains using ResFinder and Comprehensive Antibiotic Resistance Database (Perfect and Strict hits)

Gene	Antimicrobial Class	Resistance Mechanism	Isolates (ResFinder)	Isolates (CARD Perfect)	Isolates (CARD Strict)
*aac(6’)-IIc*	Aminoglycosides	Antibiotic inactivation	3424, 3442, 3443, 3412, 3415	3415	3442, 3443
*aadA*	Aminoglycosides	Antibiotic inactivation	3424, 3442, 3443, 3412, 3415	3442, 3443, 3412, 3415	3424
*sul1*	Sulfonamides	Target replacement	3424, 3442, 3443, 3412, 3415	3424, 3442, 3443, 3412, 3415	ND
*penB-4*	β-lactams	Antibiotic inactivation	ND	ND	3424, 3442, 3443, 3412, 3415, J2315, ST32
*bla*_*OXA−1043*_	β-lactams	Antibiotic inactivation	ND	ND	3424, 3442, 3443, 3412, 3415
*bla*_*OXA*−1142_	β-lactams	Antibiotic inactivation	ND	ND	J2315
*qacE∆1*	Disinfectants	Antibiotic efflux	ND	3442, 3443, 3412, 3415	3424

ND Not Detected

In the CARD database, antimicrobial resistance determinants are classified according to confidence levels: the “Perfect” algorithm identifies resistance proteins with an exact (100%) match to reference sequences, whereas the “Strict” algorithm allows for sequence variation within curated thresholds, enabling the detection of resistance gene variants or antibiotic targets altered by mutation. Accordingly, all Strict hits exhibited sequence identity values ranging from 98.88% to 99.68% and reference sequence coverage between 98.10% and 104.15%.

The AMR module of the PATRIC platform predicted 43 antimicrobial resistance genes, distributed across ten distinct mechanisms. Genes related to antibiotic targets, efflux pumps, regulation, permeability, cell wall modification, protection, activation, and target replacement were conserved in all clinical isolates and reference strains (Supplementary Table S2).

The main differences between the clinical isolates and the reference strains were observed in the antibiotic inactivation genes: *aac(6’)-Ib*, *aac(6’)-II*, and *aadA*, which were present in all clinical isolates but absent from the reference strains. Additionally, the *bla*_*OXA*_ gene (class D β-lactamase) was detected exclusively in the clinical isolates and in *B. cenocepacia* J2315 (Table 4).

**Table 4:** Putative antimicrobial resistance determinants in the five clinical isolates of *B. cenocepacia* compared with the reference strains *B. cenocepacia* J2315 and *B. cenocepacia* ST32

Gene	Product encoded	3424	3442	3443	3412	3415	J2315	ST32
*aac(6’)-Ib/aac(6’)-II*	Aminoglycoside N(6’)-acetyltransferase	P	P	P	P	P	A	A
*bla*_*OXA*_	Class D beta-lactamase	P	P	P	P	P	P	A
*aadA*	Aminoglycoside 3’’-nucleotidyltransferase	P	P	P	P	P	A	A

P present; A absent

### Mobilome Analysis

PHASTEST predicted intact prophage regions in all clinical isolates. In 3424, two intact prophages were identified: a phiE125-like element (46.5 kb; 74 CDSs; 61.33% GC) and a 31.9-kb region (38 CDSs; 63.19% GC) related to *Escherichia* phage vB_EcoM_ECOO78. Isolate 3442 harbored a phiSal3-like prophage (54.0 kb; 73 CDSs; 62.18% GC), related to *Salmonella* phage 118970_sal3, which was also detected in 3443 (53.9 kb; 71 CDSs; 62.18% GC). PhiE125-like prophages were also identified in 3412 (46.5 kb; 74 CDSs; 61.33% GC) and 3415 (47.1 kb; 70 CDSs; 61.50% GC).

Putative genomic islands (GIs) specific to the five clinical isolates were predicted with IslandViewer (IslandPath-DIMOB and SIGI-HMM) through pairwise comparison against chromosomes 1, 2, and 3 of the reference strain *B. cenocepacia* J2315. Across the five genomes, 132–152 GIs were identified, distributed across the three chromosomes and totaling approximately 2.10 Mb per genome. Within the predicted GIs of all isolates, two resistance determinants were consistently detected: dihydropteroate synthase type 2 and aminoglycoside 3″-nucleotidyltransferase ANT(3″)-Ia (aadA family). These findings were validated by manual curation using BLASTp.

Insertion sequences (IS) were identified across the genomes and belonged to three families: IS3 (ISBmu5), IS5 (ISBugl2), and IS200/IS605 (ISPa18). In 3424, 3442, 3412, and 3415, the contig carrying ISPa18 also contained the antimicrobial resistance genes *aadA1*, *qacE*, and *sul1*. The aminoglycoside resistance gene *aac(6′)-IIc* was detected in all isolates; in 3424, 3442, 3443, and 3412, it was located on contigs separate from ISPa18, whereas in 3415, both were located on the same contig (Table 5).

**Table 5:** Putative insertion sequences and associated resistance determinants identified in five *B. cenocepacia* isolates

Isolate	Contig	MGE (family/group)	Resistance determinants	Alignment coverage (%)
3424	1	ISBmu5 (IS3/IS150)	ND	99.86
	13	ISBugl2 (IS5/IS427)	ND	99.56
	132	ISPa18 (IS200/IS605/IS1341)	*sul1*, *qacE*, *aadA1*	99.33
	150	–	*aac(6′)-IIc*	NA
3442	1	ISBmu5 (IS3/IS150)	ND	99.86
	4	ISBugl2 (IS5/IS427)	ND	99.56
	107	ISPa18 (IS200/IS605/IS1341)	*sul1*, *qacE*, *aadA1*	99.33
	122	–	*aac(6′)-IIc*	NA
3443	2	ISBmu5 (IS3/IS150)	ND	99.86
	3	ISBmu5 (IS3/IS150)	ND	100.00
	7	ISBugl2 (IS5/IS427	ND	99.56
	130	ISPa18 (IS200/IS605/IS1341)	*sul1*, *qacE*, *aadA1*	99.33
	149	–	*aac(6′)-IIc*	NA
3412	15	ISBugl2 (IS5/IS427)	ND	99.56
	94	ISPa18 (IS200/IS605/IS1341)	*sul1*, *qacE*, *aadA1*	99.33
	110	–	*aac(6′)-IIc*	NA
3415	1	ISBmu5 (IS3/IS150)	ND	99.86
	11	ISBugl2 (IS5/IS427)	ND	99.56
	154	ISPa18 (IS200/IS605/IS1341)	*sul1*, *qacE*, *aadA1*,* aac(6′)-IIc*	99.33

MGE mobile genetic element; – no MGE detected; ND not detected; NA not applicable

A single conjugative plasmid (bin AD978) was identified across all *B. cenocepacia* isolates. The plasmid measured 80.16 kb on average (range 79.70–80.95 kb), with a GC content of 62.94% (range 61.66–64.85%), and was typically assembled into three contigs (range 2–4), encoding a mean of 95 CDSs (range 93–99). MOB-suite (MOB-Recon/MOB-Typer) predicted the MOBF relaxase and MPF_F mating pair formation system, consistent with a conjugative plasmid. Annotation identified *traI* (MOBF relaxase), *traD* (type IV coupling protein, T4CP), and several type IV secretion system/pilus components (*traH*, *traF*, *traN*, *traU*, *traW*, *traL*, *trbC*), along with *hns* and a transposase belonging to the IS2 family.

## Discussion

Species of the Bcc cause chronic infections in CF patients due to intrinsic multidrug resistance and high virulence, hindering eradication and worsening outcomes. *B. cenocepacia*, including epidemic strains, is associated with poorer prognosis and higher mortality, especially in cases of CS [7].

We report a novel ST (ST2424) of *B. cenocepacia* IIIA, distinct from the described clonal complexes, isolated from a CF patient who developed CS and progressed to fatal bacteremia. Comparative genomic analyses of five isolates from this patient, one from sputum and four from blood, were conducted against epidemic reference strains *B. cenocepacia* J2315 (ST28) and *B. cenocepacia* ST32, allowing us to explore genomic determinants that may contribute to persistence, systemic dissemination, and therapeutic challenges.

According to genomic similarity metrics and phylogenetic topology, the five isolates clustered more closely with *B. cenocepacia* J2315, the epidemic ET12 lineage, a pattern consistent with previous reports indicating that IIIA lineage isolates share greater homology and epidemic potential with the ET12 complex [16].

Phylogenomic analysis based on SNPs demonstrated that the five clinical *B. cenocepacia* isolates were highly related, showing low genetic divergence between the pulmonary isolate and the bloodstream isolates. This pattern is consistent with a monoclonal infection, in which a single infecting lineage persists within the host and undergoes genetic diversification over the course of infection [21]. The restricted SNP differences observed among the isolates, including the relatively higher divergence of isolate 3415, are compatible with processes of within-host microevolution, reflecting diversification from a common ancestor [22]. In this context, genomic diversification has been associated with selective pressures imposed by the pulmonary environment and prolonged antibiotic therapy during chronic infections [23].

*Burkholderia cenocepacia* exhibits high virulence potential, supported by its marked genetic diversity and the occurrence of epidemic strains with high clinical impact [24, 25]. In our isolates and in the reference strains, the presence/absence analysis based on annotation indicated that most virulence determinants previously described for *B. cenocepacia* are highly conserved in both the epidemic lineages and the evaluated isolates.

Specific variations were identified that may reflect adaptation to different host environments. In the bloodstream isolates, we identified *narG* (nitrate reductase α-subunit), linked to anaerobic metabolism and denitrification [26], and *manCcore*, which encodes GDP-mannose pyrophosphorylase and provides GDP-mannose as a precursor for O-antigen biosynthesis in the lipopolysaccharide (LPS) [27]. These differences may indicate structural and metabolic adaptations that could support persistence under low-oxygen conditions. Evidence from *Staphylococcus aureus* further supports a potential role for *narG* in influencing virulence and host immune response [28].

Additionally, variation was observed in the secretion system genes *aaiA* and *aaiB*, part of the AAI/SCI-II module first described in Enteroaggregative *Escherichia coli* (EAEC) and associated with the T6SS [29]. The *aaiA* gene was detected in all five clinical isolates and in *B. cenocepacia* ST32, whereas *aaiB* was restricted to the clinical isolates and absent from both reference strains. In EAEC, the AggR regulator activates the AAI/SCI locus, which includes *aaiA* and *aaiB* [30]. Although *aaiA* and *aaiB* in *B. cenocepacia* have not been experimentally characterized, they are part of an operon associated with the T6SS. This system has been implicated in interbacterial competition and persistence in polymicrobial environments, such as the airways of CF patients [31].

In all five isolates, the PATRIC AMR module predicted 43 resistance genes, reflecting a broad and conserved resistome characteristic of the core genome of *B. cenocepacia*. These include intrinsic mechanisms such as reduced membrane permeability, efflux pumps, and LPS modifications, recognized as essential components of the species’ multidrug-resistant phenotype [32].

Acquired resistance determinants constituted a prominent feature in these isolates. The *sul1* gene, frequently associated with class 1 integrons, was consistently identified. Although integrons are less frequently described in the Bcc [33]. This finding may indicate a recent acquisition, possibly associated with selective pressure from prolonged antimicrobial therapy, although this cannot be confirmed.

We also detected, in all clinical isolates, a new subgroup of the OXA family, *bla*_*OXA−1043*_, which encodes a class D β-lactamase recently described in *B. cenocepacia* [34]. The production of class D β-lactamases is a major public health concern as it facilitates interspecies dissemination of resistance [35]. The presence of *bla*_*OXA−1043*_ may represent an emerging mechanism of resistance.

Importantly, we identified the aminoglycoside-modifying genes *aac(6′)-Ib-like*, *aac(6′)-IIc*, and *aadA* in all five clinical isolates, whereas they were absent from the reference strains. These determinants, commonly associated with integrons and plasmids, indicate horizontal acquisition of aminoglycoside inactivation pathways, complementing the intrinsic outer membrane impermeability and resistance-nodulation-division (RND) efflux systems characteristic of Bcc [36, 37].

With respect to mobile genetic elements, in the genomes analyzed prophages accounted for approximately 0.6–1.0% of the genomic content and contained only canonical viral modules. The absence of virulence or resistance genes in the prophages observed in this study is consistent with previous reports in *B. cenocepacia*, in which phage islands from both clinical and environmental strains also lacked such determinants [38]. This pattern is consistent with the Piggyback-the-winner model, according to which, in high bacterial density environments, such as pulmonary mucus in CF, lysogenic maintenance is favored over the lytic cycle [39], although this hypothesis has not yet been directly tested in *Burkholderia*.

The presence of more than 130 genomic islands per genome (approximately 2 Mb) suggests that a considerable portion of the *B. cenocepacia* genome consists of mobile and genetically unstable regions, indicating notable genomic plasticity. This modular organization may enhance the bacterium’s capacity for gene acquisition, loss, and rearrangement through horizontal gene transfer, thereby increasing genetic variability and supporting adaptive evolutionary processes in selective environments [40] such as the CF lung. Although automated pipelines did not annotate virulence or resistance loci, manual curation confirmed the presence of *sul1* and *aadA*, indicating the integration of clinically relevant determinants within horizontally acquired regions. It should be acknowledged that IslandViewer may overestimate the number of genomic islands due to overlapping predictions among algorithms; therefore, these findings should be interpreted as estimates.

Insertion sequences were also diverse, representing the families IS200/IS605 (ISPa18), IS3 (ISBmu5 and ISButh1), and IS5 (ISBugl2). In a previous study conducted by our group, IS3 and IS5 were also identified in *B. cenocepacia* from a liver abscess in a CF patient [41]. In this study, notably, ISPa18 co-localized on the same contig with the resistance determinants *sul1*, *qacE*, *aadA1*, and *aac(6′)-IIc*. Members of the IS200/IS605 family are described as genomic hotspots for the capture of “passenger genes” and site-specific rearrangements [42, 43], which may facilitate horizontal transfer and the dissemination of resistance in clinical settings [44].

Plasmid analysis revealed a conjugative element detected in all isolates, with an average size of approximately 80 kb and about 95 CDSs. These values are consistent with those reported for *B. cenocepacia* plasmids, such as the *B. cenocepacia* J2315 strain plasmid [45]. Studies in *B. cenocepacia* describe a plasmid-encoded type IV secretion apparatus, with Tra/Trb pilus assembly subunits and a coupling protein, consistent with our findings [46, 47]. The coexistence of resistance determinants and conjugative machinery in these plasmids underscores their potential role as vehicles for horizontal gene transfer, facilitating both intra and interspecies dissemination of adaptive traits.

## Conclusion

This study provides relevant insights into a novel sequence type (ST2424) of *B. cenocepacia* genomovar IIIA, isolated from a CF patient who developed CS and progressed to fatal bacteremia. Genomic analyses revealed high similarity to the epidemic ET12 lineage and identified both conserved and distinctive virulence, resistance, and mobilome profiles, suggesting that genomic plasticity and horizontal gene transfer contribute to persistence and systemic dissemination.

It is important to highlight that all analyses in this study were conducted in silico, and experimental validation is necessary to confirm the functional roles of the identified determinants. In addition, the study was limited to samples from a single patient, which restricts the generalizability of the findings. Future studies involving larger cohorts and longitudinal sampling are recommended to evaluate the consistency and clinical relevance of the patterns observed for the new sequence type ST2424 of *B. cenocepacia* in different individuals with CF.

These findings highlight the importance of continuous genomic surveillance to monitor emerging variants and their potential clinical impact in CF, while supporting improved infection control and clinical management strategies.

## Supplementary Information

Below is the link to the electronic supplementary material.

Supplementary Material 1

Supplementary Material 2

## Data Availability

The Whole Genome Shotgun (WGS) sequencing project has been deposited in the National Center for Biotechnology Information (NCBI) under the following accession numbers: JBQRTX000000000 (3424, BioProject PRJNA1309077); JBQRTY000000000 (3442, BioProject PRJNA1309082); JBQRTZ000000000 (3443, BioProject PRJNA1309087); JBQRTV000000000 (3412, BioProject PRJNA1309062); and JBQRTW000000000 (3415, BioProject PRJNA1309070).
