# Supplementary material for: Genomic Insights into a New Burkholderia cenocepacia Sequence Type Linked to Cepacia Syndrome in Cystic Fibrosis
Source: Curr Microbiol. 2026 Jun 11;83(8):420. doi: 10.1007/s00284-026-04994-z (PMC13260241; doi:10.1007/s00284-026-04994-z)
Supplement: Supplementary file 1 — Supplementary Material 1 [file 284_2026_4994_MOESM1_ESM.docx]

Article title: Genomic insights into a new *Burkholderia cenocepacia* sequence type linked to cepacia syndrome in cystic fibrosis
Journal: Current Microbiology
Authors: Tatiane S. Xavier^1^, Felipe A. Simão^1^, Heloisa S. Rosa^1^, Jade C. S. Colomer^1^, Renata W. Cohen^2^, Tânia W. Folescu^2^, Ana Paula D’A. Carvalho-Assef^3^, Robson S. Leão^1^ and Elizabeth A. Marques^1*^

**Affiliation:** ^1^ Laboratório de Microbiologia da Fibrose Cística, Departamento de Microbiologia, Imunologia e Parasitologia, Faculdade de Ciências Médicas, Universidade do Estado do Rio de Janeiro, Rio de Janeiro, Brazil.

^2^ Instituto Nacional de Saúde da Mulher, da Criança e do Adolescente Fernandes Figueira, Centro de Referência para Crianças e Adolescentes com Fibrose Cística – Ministério da Saúde, Fundação Oswaldo Cruz, Rio de Janeiro, Brazil.

^3^ Laboratório de Bacteriologia Aplicada à Saúde Única e Resistência Antimicrobiana, Instituto Oswaldo Cruz, Rio de Janeiro, Brazil.
**Corresponding author:** Elizabeth A. Marques (marbe@uerj.br)

| **Supplementary table S1** Putative virulence genes from the five *Burkholderia cenocepacia* clinical isolates compared with the reference strains *B. cenocepacia* J2315 and *B. cenocepacia* ST32 | | | | | | | | | |
| --- | --- | --- | --- | --- | --- | --- | --- | --- | --- |
| **Gene** | **Product encoded** | **3424** | **3442** | **3443** | **3412** | **3415** | **J2315** | **ST32** | |
| **Adherence** | | | | | | | | |  |
| *boaA, boaB* | Type IV pili | P | P | P | P | P | P | P | |
| *pilA, pilB, pilC, pilD* |  | P | P | P | P | P | P | P | |
| *flpF* | Flp type IV pili | P | P | P | P | P | P | P | |
| **Antiphagocytosis** | | | | | | | | |  |
| *manC* | Capsule I | P | P | P | P | P | P | P | |
| *wcbA, wcbB, wcbC, wcbD wcbO, wcbP, wcbQ, wcbR, wcbS, wcbT* |  | P | P | P | P | P | P | P | |
| *wzm* |  | P | P | P | P | P | P | P | |
| *wzt2* |  | P | P | P | P | P | P | P | |
| *wbjD/wecB* | Capsular polysaccharide | P | P | P | P | P | P | A | |
| **Invasion** | | | | | | | | |  |
| *cheA, cheB, cheD, cheR, cheW, cheY, cheY1, cheZ* | Flagella | P | P | P | P | P | P | P | |
| *flgA, flgB, flgC, flgD, flgE flgF, flgG, flgH, flgI, flgJ flgK, flgL, flgM, flgN* |  | P | P | P | P | P | P | P | |
| *flhA, flhB, flhF, flhG* |  | P | P | P | P | P | P | P | |
| *fliA, fliC, fliD, fliE, fliF, fliG fliH*  *fliI, fliJ, fliK, fliL, fliM, fliN, fliO, fliP, fliQ, fliR, fliS* |  | P | P | P | P | P | P | P | |
| *motA, motB* |  | P | P | P | P | P | P | P | |
| *Tsr* |  | P | P | P | P | P | P | P | |
| *pmlR/bspR1* | Quorum sensing | P | P | P | P | P | P | P | |
| *pmlI/bspI1* |  | P | P | P | P | P | P | P | |
| **Secretion system** | | | | | | | | |  |
| *bprA,* | Bsa T3SS | P | P | P | P | P | P | P | |
| *bsaQ* |  | P | P | P | P | P | P | P | |
| *aaiA* | AAI/SCI-II T6SS | P | P | P | P | P | A | P | |
| *aaiB* |  | P | P | P | P | P | A | A | |
| *pscS* | TTSS | P | P | P | P | P | P | P | |
| *vscR* | T3SS1 | P | P | P | P | P | P | P | |
| *bscN* | Type III secretion system | P | P | P | P | P | P | P | |
| **Anaerobic respiration** | | | | | | | | |  |
| *narG* | Nitrate reductase subunit alpha | A | P | P | P | P | A | A | |
| **Biofilm formation** | | | | | | | | |  |
| *adeG* | AdeFGH efflux pump/transport autoinducer | P | P | P | P | P | P | P | |
| **Cell surface components** | | | | | | | | |  |
| *sugC* | Trehalose-recycling ABC transporter | P | P | P | P | P | P | P | |
| **Efflux pump** | | | | | | | | |  |
| *acrB* | AcrAB | P | P | P | P | P | P | P | |
| *farB* | FarAB | P | P | P | P | P | P | P | |
| *mtrD* | MtrCDE | P | P | P | P | P | P | P | |
| **Enzyme** | | | | | | | | |  |
| *plc* | Phospholipase C | P | P | P | P | P | P | P | |
| **Immune evasion** | | | | | | | | |  |
| *gtrB* | LPS glucosylation | P | P | P | P | P | P | P | |
| *manCcore* | LPS | A | P | A | P | P | A | A | |
| **Iron uptake** | | | | | | | | |  |
| *fptA* | Pyochelin receptor | P | P | P | P | P | P | P | |
| *pchF* | Pyochelin synthetase | P | P | P | P | P | A | P | |
| *pchB pchC pchD pchE pchF pchG pchH pchI pchR* | Pyochelin | P | P | P | P | P | P | P | |
| *pvdF* | Pyoverdine | P | P | P | P | P | P | P | |
| *ccmE, ccmF* | Cytochrome c maturation locus | A | A | A | A | A | A | P | |

Supplementary table S1 Putative virulence genes from the five *Burkholderia cenocepacia* clinical isolates compared with the reference strains *B. cenocepacia* J2315 and *B. cenocepacia* ST32. The presence or absence of each gene was determined based on data obtained from the VFDB (Virulence Factor Database). Genes are grouped according to functional categories such as adherence, antiphagocytosis, invasion, secretion systems, biofilm formation, and iron uptake; P present; A absent.
