# Supplementary material for: Genomic Insights into a New Burkholderia cenocepacia Sequence Type Linked to Cepacia Syndrome in Cystic Fibrosis
Source: Curr Microbiol. 2026 Jun 11;83(8):420. doi: 10.1007/s00284-026-04994-z (PMC13260241; doi:10.1007/s00284-026-04994-z)
Supplement: Supplementary file 2 — Supplementary Material 2 [file 284_2026_4994_MOESM2_ESM.docx]

Article title: Genomic insights into a new *Burkholderia cenocepacia* sequence type linked to cepacia syndrome in cystic fibrosis
Journal: Current Microbiology
Authors: Tatiane S. Xavier^1^, Felipe A. Simão^1^, Heloísa S. Rosa^1^, Jade C. S. Colomer^1^, Renata W. Cohen^2^, Tânia W. Folescu^2^, Ana Paula D’A. Carvalho-Assef^3^, Robson S. Leão^1^ and Elizabeth A. Marques^1*^

**Affiliation:** ^1^ Laboratório de Microbiologia da Fibrose Cística, Departamento de Microbiologia, Imunologia e Parasitologia, Faculdade de Ciências Médicas, Universidade do Estado do Rio de Janeiro, Rio de Janeiro, Brazil.

^2^ Instituto Nacional de Saúde da Mulher, da Criança e do Adolescente Fernandes Figueira, Centro de Referência para Crianças e Adolescentes com Fibrose Cística – Ministério da Saúde, Fundação Oswaldo Cruz, Rio de Janeiro, Brazil.

^3^ Laboratório de Bacteriologia Aplicada à Saúde Única e Resistência Antimicrobiana, Instituto Oswaldo Cruz, Rio de Janeiro, Brazil.
**Corresponding author:** Elizabeth A. Marques (marbe@uerj.br)

| **Supplementary table S2** Putative antimicrobial resistance determinants in five *Burkholderia cenocepacia* clinical isolates compared with those annotated in the reference strains *B. cenocepacia* J2315 and *B. cenocepacia* ST32 | | | | | | | | |
| --- | --- | --- | --- | --- | --- | --- | --- | --- |
| **Gene** | **Product encoded** | **3424** | **3442** | **3443** | **3412** | **3415** | **J2315** | **ST32** |
| **Antibiotic inactivation enzyme** | | | | | | | | |
| *aac(6')-Ib/aac(6')-II* | Aminoglycoside N(6')-acetyltransferase | P | P | P | P | P | A | A |
| *aph(3')-II/aph(3')-XV* | Aminoglycoside 3'-phosphotransferase | P | P | P | P | P | P | P |
| *penA* | Class A beta-lactamase | P | P | P | P | P | P | P |
| *ampC* | Class C beta-lactamase | P | P | P | P | P | P | P |
| OXA | Class D beta-lactamase | P | P | P | P | P | P | A |
| *aadA* | Aminoglycoside 3''-nucleotidyltransferase | P | P | P | P | P | A | A |
|  | Aminoglycoside 6-phosphotransferase, putative | P | P | P | P | P | P | P |
| **Antibiotic target in susceptible species** | | | | | | | | |
| *MurA* | UDP-N-acetylglucosamine 1-carboxyvinyltransferase | P | P | P | P | P | P | P |
| *EF-G, EF-Tu* | Translation elongation | P | P | P | P | P | P | P |
| *Dxr* | 1-deoxy-D-xylulose 5-phosphate reductoisomerase | P | P | P | P | P | P | P |
| *gyrA,gyrB* | DNA gyrase | P | P | P | P | P | P | P |
| *Iso-tRNA* | Isoleucyl-tRNA synthetase | P | P | P | P | P | P | P |
| *rpsJ, rpsL* | SSU ribosomal protein S10p e *S12p* | P | P | P | P | P | P | P |
| *rpoB, rpoC* | DNA-directed RNA polymerase beta' subunit | P | P | P | P | P | P | P |
| *Alr* | Alanine racemase | P | P | P | P | P | P | P |
| *Ddl* | D-alanine--D-alanine ligase | P | P | P | P | P | P | P |
| *kasA* | 3-oxoacyl-[acyl-carrier-protein] synthase, KASII | P | P | P | P | P | P | P |
| *Rho* | Transcription termination factor Rho | P | P | P | P | P | P | P |
| *folA, Dfr* | Dihydrofolate reductase | P | P | P | P | P | P | P |
| *folP* | Dihydropteroate synthase | P | P | P | P | P | P | P |
| **Efflux pump conferring antibiotic resistance** | | | | | | | | |
| *EmrAB-TolC* | Multidrug efflux system EmrAB-OMF, inner-membrane proton/drug antiporter EmrB (MFS type) | P | P | P | P | P | P | P |
| *MexXY-OMP* | Multidrug efflux system, membrane fusion component | P | P | P | P | P | P | P |
| *MdtABC-TolC* | Multidrug efflux system MdtABC-TolC, inner-membrane proton/drug antiporter MdtC (RND type) | P | P | P | P | P | P | P |
| *MdtABC-OMF, EmrAB-OMF* | Outer membrane factor (OMF) lipoprotein | P | P | P | P | P | P | P |
| *MacB* | Macrolide export ATP-binding/permease protein | P | P | P | P | P | P | P |
| *MacA* | Macrolide-specific efflux | P | P | P | P | P | P | P |
| *ceoB* | RND efflux system, inner membrane transporter | P | P | P | P | P | P | P |
| **Regulator modulating expression of antibiotic resistance genes** | | | | | | | | |
| *hns* | DNA-binding protein | P | P | P | P | P | P | P |
| *OxyR* | Hydrogen peroxide-inducible genes activator | P | P | P | P | P | P | P |
| **Protein modulating permeability to antibiotic** | | | | | | | | |
| *OprB, OprD* | Outer membrane low permeability porin | P | P | P | P | P | P | P |
| **Protein altering cell wall charge conferring antibiotic resistance** | | | | | | | | |
| *PgsA* | CDP-diacylglycerol-glycerol-3-phosphate 3-phosphatidyltransferase | P | P | P | P | P | P | P |
| *GdpD* | Glycerophosphoryl diester phosphodiesterase | P | P | P | P | P | P | P |
| **Gene conferring resistance via absence** | | | | | | | | |
| *gidB* | 16S rRNA (guanine(527)-N(7))-methyltransferase | P | P | P | P | P | P | P |
| **Antibiotic activation enzyme** | | | | | | | | |
| *KatG* | Catalase-peroxidase | P | P | P | P | P | P | P |
| **Antibiotic target protection protein** | | | | | | | | |
| *BcrC* | Undecaprenyl-diphosphatase, conveys bacitracin resistance | P | P | P | P | P | P | P |
| **Antibiotic target replacement protein** | | | | | | | | |
| *fabV* | Enoyl-[acyl-carrier-protein] reductase [NADH] | P | P | P | P | P | P | P |

Supplementary Table S2 Putative antimicrobial resistance determinants in five *Burkholderia cenocepacia* clinical isolates compared with reference strains *B. cenocepacia* J2315 and *B. cenocepacia* ST32. The presence or absence of each resistance gene was determined using data retrieved from the PATRIC database (Pathosystems Resource Integration Center). Categories were grouped according to the resistance mechanism: antibiotic inactivation enzymes, target alteration, efflux pumps, and permeability or regulatory proteins; P present; A absent.
